# Buckling of regular, chiral and hierarchical honeycombs under a general macroscopic stress state

**DOI:** 10.1098/rspa.2013.0856

**Published:** 2014-07-08

**Authors:** Babak Haghpanah, Jim Papadopoulos, Davood Mousanezhad, Hamid Nayeb-Hashemi, Ashkan Vaziri

**Affiliations:** Department of Mechanical and Industrial Engineering, Northeastern University, Boston, MA, USA

**Keywords:** instability, cellular structure, beam-column, in-plane loading

## Abstract

An approach to obtain analytical closed-form expressions for the macroscopic ‘buckling strength’ of various two-dimensional cellular structures is presented. The method is based on classical beam-column end-moment behaviour expressed in a matrix form. It is applied to sample honeycombs with square, triangular and hexagonal unit cells to determine their buckling strength under a general macroscopic in-plane stress state. The results were verified using finite-element Eigenvalue analysis.

## 1. Introduction

Low-density cellular materials have found widespread application for energy absorption, structural protection and as the core of lightweight sandwich panels. However, compressive loads can cause the cell walls to buckle, which limits their strength. Collapse of the cellular material due to the buckling becomes more likely as relative density is reduced [1]. Additionally, microscopic instability patterns could be deliberately used as a technique for induction or modification of microscopic, periodicity-dependent structural or surface properties such as chirality [2–4], wave propagation and phononic properties [5–11], optical characteristics [12–15], modulated nano patterns [16], hydrophobicity [17–20] or generating macroscopic responses [21,22] in periodic solids. We thus see value in a general technique to predict the instability of regular cellular structures subjected to a general macroscopic state of stress.

Previous studies on the buckling of periodic cellular structures were largely numerical or experimental. Ohno *et al.* [23] suggested a numerical method to study the buckling of cellular solids subjected to macroscopically uniform compression using a homogenization framework of the updated Lagrangian type. Triantafyllidis & Schraad [24] studied the onset of failure in honeycombs under general in-plane loading using finite-element (FE) discretization of Bloch wave theory. Abeyaratne & Triantafyllidis [25] associated instability of periodic solids with the loss of ellipticity in the incremental response of homogenized deformation behaviour. A full-scale FE study of intact and damaged hexagonal honeycombs has been presented by Guo & Gibson [26]. Several experimental studies have concerned the buckling of different cellular structures including hexagonal and circular honeycombs [27–33].

**Figure 1.**
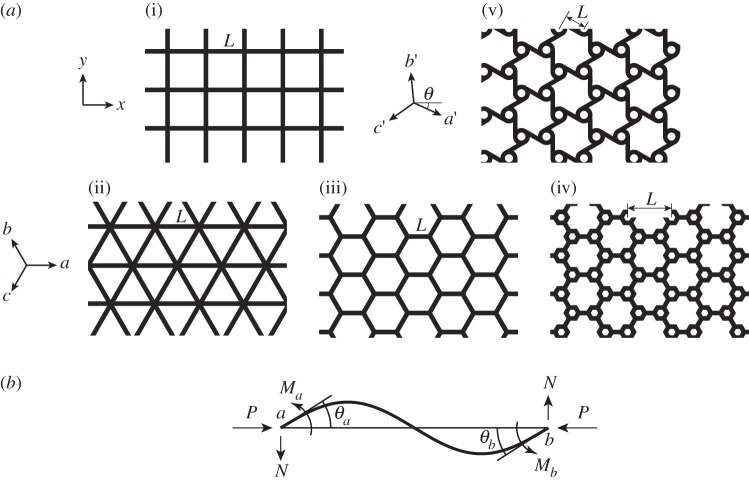
(*a*) Types of lattice structures analysed by the beam-column matrix method: (i) square grid (§4*a*); (ii) triangular grid (isogrid—§4*b*); (iii) hexagonal honeycomb (§4*c*); (iv) hierarchical hexagonal honeycomb (electronic supplementary material, appendix C) and (v) tri-chiral honeycomb (electronic supplementary material, appendix D). The angle *θ* gives the orientation of straight walls in the tri-chiral lattice. (*b*) The angles of rotation and moments of the ends (positive when counter clockwise) in a beam-column.

This study does not address localized buckling patterns in cellular materials (e.g. row wise) that can occur due to the presence of imperfections [24], boundary effects [34] or material nonlinearity [32]. However, the collapse surfaces obtained here for periodic buckling patterns in perfect cellular materials provide an upper bound for the onset of failure in the corresponding actual materials that contain inevitable imperfections in their underlying microstructures [24]. The analytical method presented here is inspired by research of Gibson *et al.* [35] on the stability of regular hexagonal honeycombs under in-plane macroscopic biaxial stress parallel to material symmetry directions (i.e. *x* and *y* in figure 1) using the beam-column solution of Manderla & Maney [36,37], as presented by Timoshenko & Gere [38]. In §2, we express the beam-column result in matrix form, to develop analytical closed-form expressions of the microscopic buckling strength for periodic beam structures under a general in-plane loading. In §3, the FE analysis used to verify the analytical results is explained. In §4, the proposed analytical approach is used to predict buckling of regular square, triangular and hexagonal honeycombs as shown in figure 1. (The illustrated hierarchical and tri-chiral hexagonal honeycombs are treated in the electronic supplementary material, appendices for the sake of brevity but the results are summarized here.) The periodic square grid is also shown to buckle according to long-wave macroscopic patterns under certain boundary conditions [39], a phenomenon observed in some three-dimensional foams [40]. Therefore, we also calculate its long-wave buckling strength under arbitrary loading as the wavelength approaches infinity. Conclusions are drawn in §5.

## 2. Method

A unit cell, or a *primitive cell* in classical physics, is the smallest structural unit, by assembling which the undeformed geometrical and loading patterns in a tessellated solid are recreated. When cellular solids are subjected to loading, buckling may occur in cell walls and edges, with a deformation pattern repeated on finite wavelengths. This kind of buckling, known as microscopic buckling, is repeated over wavelengths which can be longer than the unit cell [41]. Such mode-size repeating patterns in the buckled structure are widely known as *representative volume elements* (RVEs) and might be different under various macroscopic loading conditions applied to the structure [42]. In order to obtain the periodicity of a buckled tessellated structure, various approaches including Bloch wave analysis [24,43,44], block-diagonalization [45], Eigenvalue analysis on RVEs of progressively increasing size [43,46], full-scale FE analysis [24,26,30,47] and experimental investigations [28,29,32,33] have been used.

The methods proposed here for obtaining closed-form expressions of macroscopic buckling strength are based on ‘assumed’ buckling modes, providing the size of RVE and its overall buckled geometry. Fortunately, the number of different buckling modes observed for a cellular structure under different macroscopic loadings is usually small. For instance, just two microscopic buckling patterns are found in the literature for square, triangular and hexagonal honeycombs under various loading conditions.

### (a) Beam-column end moments

The beam-column formula is a classical approach linking the end rotations of an axially loaded beam to its end moments. This approach has been used to obtain the buckling strength of cellular structures under simplified loading conditions, including uniaxial and biaxial loadings [35,48]. However, the complexity of an arbitrary stress state complicates the beam-column equations, especially for larger RVEs with higher nodal connectivity. In this section, we present the characteristic nonlinear beam-column equations in a matrix form, allowing a more systematic calculation of the buckling strength. The *symbolic* calculation tool in MATLAB is then used to obtain closed-form expressions of buckling strength.

For a single beam connecting nodes *a* and *b* under axial compressive force *P* and subjected to the two counter clockwise end couples *M*_*a*_ and *M*_*b*_, the end rotations *θ*_*a*_ and *θ*_*b*_ (positive when counter clockwise) relative to the line joining the displaced end nodes (figure 1*b*) can be obtained through the beam-column relations as [38]

2.1 $$\begin{array}{lll} & \theta_{a} & =+\frac{M_{a}l}{EI}\Psi (q)-\frac{M_{b}l}{EI}\Phi (q)\\ \mathrm{and} & \theta_{b} & =-\frac{M_{a}l}{EI}\Phi (q)+\frac{M_{b}l}{EI}\Psi (q). \end{array}\}$$

Here, Φ(q)=(1/sin⁡q−1/q)/q and Ψ(q)=(1/q−1/tan⁡q)/q are nonlinear functions of the non-dimensional loading parameter *q* defined as $q=l\sqrt{P/(EI)}$, where *P* is the beam axial force (positive when compressive), *l* is the beam length and *EI* is the beam flexural rigidity. The functions *Φ* and *Ψ* can be approximated by even-order expansions, climbing monotonically from *Φ*=1/6 and *Ψ*=1/3 when *q*=0 to infinity when *q*=*π*.) Taking the rigid body rotation *β* of the beam (i.e. the line joining the beam ends) as an additional degree of freedom, the set of three boundary conditions (i.e. two on beam end displacements or moments, and one on beam rotation *β*) and two beam-column relations given in equation (2.1) can be expressed in the following matrix form to obtain the buckling of a single beam under axial loading:

2.2 $${\left[A\right]}_{5\times 5}{\left[\begin{array}{c} M_{a}l/EI\\ M_{b}l/EI\\ \theta_{a}\\ \theta_{b}\\ \beta_{ab} \end{array}\right]}_{5\times 1}={\left[B\right]}_{5\times 1}.$$

Here, matrix *A* is termed the system's *characteristic matrix*, and the condition |*A*|=0 gives the critical value of *q* and hence the buckling load *P*_*c*_. Vector *B* contains any loading terms appearing in the problem which do not explicitly include any beam end moments, beam end rotations or axial loads. From the physical point of view, components of vector *B* are terms which only cause a static deflection without buckling (e.g. a transverse load component applied to the tip of a cantilevered axially loaded beam-column). Vector *B* does not affect the magnitude of the critical loads obtained from Eigenvalue buckling analysis (electronic supplementary material, appendix A shows how this matrix representation can be used for solving standard single-column buckling problems.)

For the case of a cellular structure's RVE consisting of several beams and connecting nodes, the relationships between nodal rotations and end moments for each beam, and also the boundary conditions, can be assembled in the following general matrix form:

2.3 $${\left[A\right]}_{n\times n}{\left[\begin{array}{c} Ml/EI\\ \theta \\ \beta \\ \vdots \end{array}\right]}_{n\times 1}={\left[B\right]}_{n\times 1}.$$

The condition |*A*|=0 gives the relationship between the magnitudes of axial load for the beams inside a RVE that cause it to become unstable. When [*B*]=0 or small, this instability condition (i.e. |*A*|=0) translates to the possibility of unlimited increase in values of nodal (or beam) rotations or beam end moments at finite, fixed values of beam axial loads. When [*B*] is non-zero and sufficiently large, the bifurcation response is suppressed by a static, stable deformation of the structure. Therefore, while the condition |*A*|=0 is mathematically sufficient to predict an Eigenvalue buckling, both |*A*|=0 and [*B*]=0 have to be simultaneously satisfied for an ideal bifurcation in the load–displacement response. Analysis of the existing experimental data [49] on rubber honeycombs with hexagonal cells marks the possibility of buckling at non-zero, but relatively small, values of [*B*].

## 3. Finite-element simulations

FE Eigenvalue buckling analysis was performed to validate the closed-form estimates of buckling strength. Two-dimensional elastic beam element models of the RVE were constructed using the FE software ABAQUS. These small RVE models were subjected to external loads derived from static analysis of the structure subjected to arbitrary states of macroscopic stress. (For the case of the statically indeterminate triangular grid, conditions from the periodicity of the unit cell are additionally required [50].) Rotation and displacement constraints following from the periodicity of the RVE for each buckling mode were also applied to the outer nodes. A mesh sensitivity analysis was carried out to ensure that the numerical solutions are mesh-independent. As there is little data on the in-plane buckling behaviour of hierarchical and tri-chiral honeycombs, large-scale FE models of these structures were developed initially, to determine their modes of buckling and the RVE size (see §2 for criteria for determining the RVE).

## 4. Buckling of cellular structures under a general macroscopic stress state

As examples of the method outlined in §2, the buckling stresses of some two-dimensional cellular structures including hexagonal honeycombs and square and triangular grids (figure 1) are computed.

### (a) Square honeycombs

**Figure 2.**
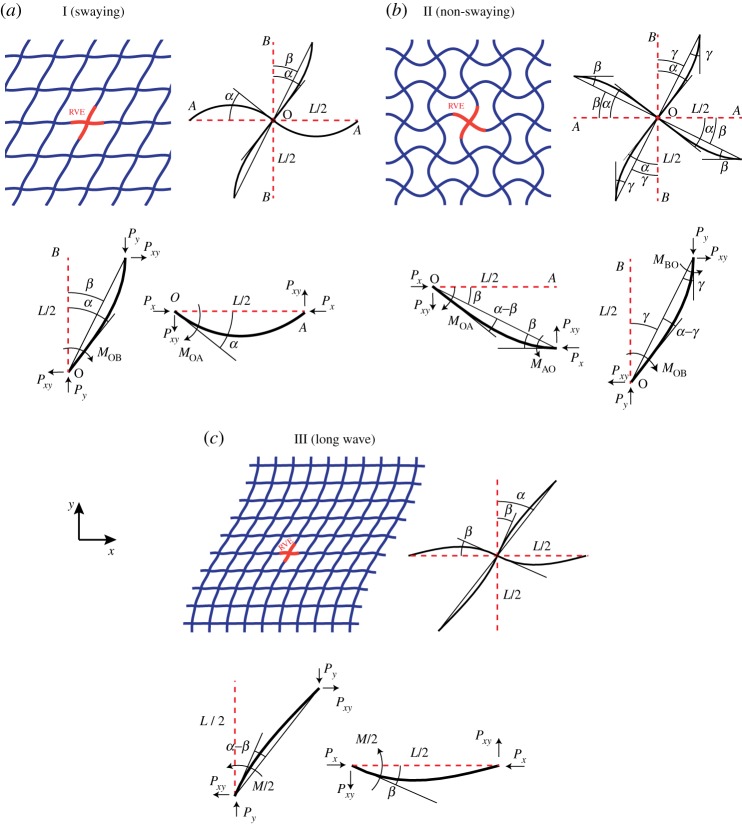
(*a*–*c*) The microscopic buckling modes for the square grid. The RVE in each mode is denoted by bold lines. Notations for the beam and nodal rotation in the RVE for each mode are given in the top right. Free-body diagrams of the RVE beam-elements are given in the bottom. (Online version in colour.)

Wah [51] first studied the stability of finite-size rectangular gridworks for both in-plane and out-of-plane loadings. Under uniaxial compressive loading parallel to cell walls, the strength of a square grid according to the sway of the axially loaded cell walls (figure 2*a*) was estimated as *S*_*c*_/*E*_*s*_=(*π*^2^/12)*(*t*/*L*)^3^, where cell walls of length *L* are treated as side-swaying columns with fixed slope at both ends [52]. This is an upper bound estimate of the actual buckling strength of square grid structure since it ignores structural rotational compliance that actually permits slope change. Fan *et al.* [48] calculated the uniaxial buckling strength of square honeycombs for the two numerically observed microscopic buckling patterns, swaying and non-swaying modes, using the beam-column method (figure 2*a*,*b*). They expressed the uniaxial buckling strengths of the structure as *S*_*Ic*_/*E*_*s*_=((0.76*π)^2^/12)*(*t*/*L*)^3^ and *S*_IIc_/*E*_*s*_=((1.292*π)^2^/12)*(*t*/*L*)^3^, with the mode I strength being less than the upper bound solution from [52]. Also, the long-wave bifurcation in square honeycombs under in-plane loading was previously studied through a two-scale theory of the updated Lagrangian type by Ohno *et al.* [39]. They showed that unlike hexagonal honeycombs, the long-wave buckling patterns in periodic square honeycombs could occur at loads lower than the critical loads corresponding to some microscopic buckling patterns. According to their analysis, by increasing the wavelength to infinity the buckling strength under either uniaxial or biaxial loadings parallel to material directions (i.e. *x* and *y* in figure 1) would approach *S*_*c*_/*E*_*s*_=(1/2)*(*t*/*L*)^3^ (where the structures buckle due to the higher principal stress and ignore the lower). The buckling wavelength for long-wave buckling patterns is shown to be dependent on the size of the finite structure and also on the boundary conditions applied to a finite structure [39].

Here, for the first time we derive closed-form relations for the microscopic buckling patterns in the square grid, as well as the long-wave buckling patterns under general in-plane loading conditions. For example, we show that under equi-biaxial (*S*^*x*^=*S*^*y*^) macroscopic loading the buckling strength (*S*^*x*^) of a square grid is $S_{c}^{x}/E_{s}=0.453*{\left(t/L\right)}^{3}$; for pure shear (*S*^*x*^=−*S*^*y*^,*S*^*x*^>0) it is $S_{c}^{x}/E_{s}=0.493*{\left(t/L\right)}^{3}$.

#### (i) Mode I (swaying)

Sway buckling shown in figure 2*a* is a shear deformation with only one set of beams changing their mean orientation, as expected for an overloaded building frame where the ground connection keeps the horizontal beams level. By making cuts through cell wall midpoints, internal reaction forces per unit depth (positive when compressive) in the beams' axial and transverse directions, denoted *P*_*x*_, *P*_*y*_ and *P*_*xy*_ in figure 2*a*, are obtained for a square grid of beam length *L* [1]

4.1 $$\left[\begin{array}{c} P_{x}\\ P_{y}\\ P_{xy} \end{array}\right]=-L\left[\begin{array}{ccc} 1 & 0 & 0\\ 0 & 1 & 0\\ 0 & 0 & 1 \end{array}\right]\;\left[\begin{array}{c} \sigma_{xx}\\ \sigma_{yy}\\ \tau_{xy} \end{array}\right].$$

Consequently, the non-dimensional *x*- and *y*-direction beam axial loading parameters *q*_*x*_ and *q*_*y*_ are obtained as $q_{x}=L/2*\sqrt{P_{x}/(EI)}=\text{i}\sqrt{3{\bar{\sigma }}_{xx}}$ and $q_{y}=L/2*\sqrt{P_{y}/(EI)}=\text{i}\sqrt{3{\bar{\sigma }}_{yy}}$, where $\text{i}=\sqrt{-1}$ and the stresses are normalized according to $\bar{\sigma }=(\sigma /E)/{\left(t/L\right)}^{3}$.

For the swaying mode of buckling due primarily to compressive *y* stress, the RVE consists of horizontal beam type OA and vertical beam type OB, as shown in figure 2*a*. These beams, respectively, experience end moments *M*_OA_ and *M*_OB_ at node O, and because of 180^°^ rotational symmetry at the outer nodes *A* and *B* are moment-free there. The unknown rotation of node O is defined as −*α*. Then the beam-column and equilibrium relations of the different beam types OA and OB can be expressed in the matrix form given below. The first row expresses the beam-column relation for rotation −*α* of end O of OA, where there is a moment at O only, and overall beam rotation *β* is zero. The second row, for the relative rotation *β*−*α* of end O of beam OB, again has a moment at O only, but this time rotation *β* is non-zero. The third row corresponds to the moment equilibrium of node O, and the last row expresses the moment equilibrium of beam OB about point O

4.2 $$\underset{A}{\underline{\left[\begin{array}{cccc} -\Psi (q_{x})/2 & 0 & 1 & 0\\ 0 & -\Psi (q_{y})/2 & 1 & -1\\ 1 & 1 & 0 & 0\\ 0 & -1 & 0 & -2q_{y}^{2} \end{array}\right]}}\;\left[\begin{array}{c} M_{\mathrm{OA}}L/(EI)\\ M_{\mathrm{OB}}L/(EI)\\ \alpha \\ \beta \end{array}\right]=\underset{B}{\underline{\left[\begin{array}{c} 0\\ 0\\ 0\\ -P_{xy}L^{2}/(2EI) \end{array}\right]}}.$$

For buckling to occur, i.e. for *α* and *β* to spontaneously take on non-zero values, the determinant of matrix *A* must vanish due to its dependence on the loads, i.e. *q*_*x*_ and *q*_*y*_. The relation for the threshold of in-plane instability in a square grid according to the swaying mode (mode I) of buckling with the *x*-direction beams held level is therefore

4.3a $$\frac{1}{q_{x}^{2}}\left(1-\frac{q_{x}}{\tan(q_{x})}\right)-\frac{1}{q_{y}^{2}}\left(\frac{q_{y}}{\tan(q_{y})}\right)=0.$$

**Figure 3.**
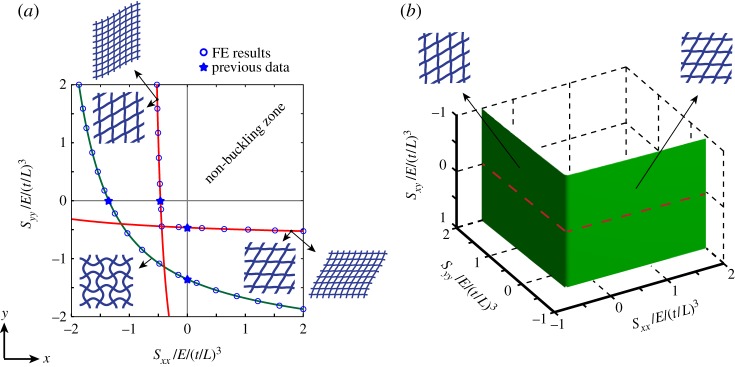
(*a*) Buckling of square honeycomb under *x*–*y* biaxial loading according to swaying, non-swaying and long-wave modes of buckling. (*b*) The buckling collapse surface for the square grid in the *σ*_*xx*_−*σ*_*yy*_−*τ*_*xy*_ stress space, allowing prediction of buckling strength under a general in-plane state of stress. The left- and right-hand sides of the instability surface correspond to the sway of horizontal and vertical beams. The condition [*B*]=0 is marked by dashed lines on the instability surface. (Online version in colour.)

This relation is plotted in figure 3*a*, where it can be seen that *x* tension is very slightly protective against sway of *y*-direction beams due to *y* compression. A second relation is required for the sway of *x*-direction beams

4.3b $$\frac{1}{q_{y}^{2}}\left(1-\frac{q_{y}}{\tan(q_{y})}\right)-\frac{1}{q_{x}^{2}}\left(\frac{q_{x}}{\tan(q_{x})}\right)=0.$$

Note that *P*_*xy*_ is irrelevant to the question of buckling, since it appears only on the right-hand side of equation (4.2). However, it leads to unbounded *α* and *β* as matrix *A* approaches singularity. Substituting from $q_{x}=\text{i}\sqrt{3{\bar{\sigma }}_{xx}}$ and $q_{y}=\text{i}\sqrt{3{\bar{\sigma }}_{yy}}$ into equations (4.3) yield the following relations for the first-mode buckling of a square grid

4.4a $${\bar{\sigma }}_{yy}\left(1-\sqrt{3{\bar{\sigma }}_{xx}}\;\text{coth}\left(\sqrt{3{\bar{\sigma }}_{xx}}\right)\right)-{\bar{\sigma }}_{xx}\left(\sqrt{3{\bar{\sigma }}_{yy}}\;\text{coth}\left(\sqrt{3{\bar{\sigma }}_{yy}}\right)\right)=0$$

and

4.4b $${\bar{\sigma }}_{xx}\left(1-\sqrt{3{\bar{\sigma }}_{yy}}\;\text{coth}\left(\sqrt{3{\bar{\sigma }}_{yy}}\right)\right)-{\bar{\sigma }}_{yy}\left(\sqrt{3{\bar{\sigma }}_{xx}}\;\text{coth}\left(\sqrt{3{\bar{\sigma }}_{xx}}\right)\right)=0.$$

#### (ii) Mode II (non-swaying)

Non-swaying buckling involves *x* and/or *y* compressive buckling with no macroscopic shearing deformation. This requires cooperative buckling in adjacent cells to minimize energy. The same definitions of *P*_*x*_, *P*_*y*_, *P*_*xy*_, *q*_*x*_ and *q*_*y*_ are used as above. Figure 2*b* shows the non-swaying mode (mode II) with the structural RVE indicated by red lines, as well as the free-body diagram of the RVE. Now the beam midpoints are not moment-free, so both *M*_OA_ and *M*_*AO*_ come into play. (Note the altered sign convention for *M*_OA_.)

The set of beam-column relations can be expressed in the following matrix form. The first two rows express moment equilibrium of beams OA and OB about point O. Rows three to six represent the beam-column relations for relative rotation of both ends of beams OA and OB. The last row expresses the moment equilibrium of node O

4.5 $$\left[\begin{array}{ccccccc} -1 & 1 & 0 & 0 & 0 & -2q_{x}^{2} & 0\\ 0 & 0 & -1 & 1 & 0 & 0 & -2q_{y}^{2}\\ \frac{\Phi (q_{x})}{2} & \frac{\Psi (q_{x})}{2} & 0 & 0 & 0 & -1 & 0\\ \frac{\Psi (q_{x})}{2} & \frac{\Phi (q_{x})}{2} & 0 & 0 & -1 & 1 & 0\\ 0 & 0 & \frac{\Phi (q_{y})}{2} & \frac{\Psi (q_{y})}{2} & 0 & 0 & -1\\ 0 & 0 & \frac{\Psi (q_{y})}{2} & \frac{\Phi (q_{y})}{2} & -1 & 0 & 1\\ 1 & 0 & 1 & 0 & 0 & 0 & 0 \end{array}\right]\;\left[\begin{array}{c} M_{\mathrm{OA}}L/(EI)\\ M_{\mathrm{AO}}L/(EI)\\ M_{\mathrm{OB}}L/(EI)\\ M_{\mathrm{BO}}L/(EI)\\ \alpha \\ \beta \\ \gamma \end{array}\right]\;\left[\begin{array}{c} -P_{xy}L^{2}/(2EI)\\ +P_{xy}L^{2}/(2EI)\\ 0\\ 0\\ 0\\ 0\\ 0 \end{array}\right].$$

Setting the determinant of the characteristic matrix to zero results in $q_{x}\cot(q_{x})+q_{y}\cot(q_{y})=0$. Since this relation is symmetric in *x* and *y* (figure 3*a*), no additional expression is needed. Later we will see that the magnitudes of stresses satisfying this relationship are always greater than the stresses required for the swaying mode of buckling, described by equations (4.4). Therefore, the non-swaying buckling mode is never the preferred mode of buckling under macroscopic stress state.

#### (iii) Mode III (long-wave)

The long-wave mode of buckling was previously studied by Ohno and co-workers through FE discretization of a two-scale theory of the updated Lagrangian type [39,53,54]. They estimated the uniaxial onset stress of long-wave buckling for the square grid as *S*^*x*^_LW*c*_/*E*_*s*_=0.5*(*t*/*L*)^3^. Here, the long-wave buckling strength of a square grid under a general stress state is sought as the wavelength approaches infinity, using the beam-column method. Figure 2*c* shows a deformed structure according to this buckling pattern, where the RVE is defined as a cross-shaped unit connecting four adjacent beam centres. Note that under the long-wave mode shown in this figure all vertical lines deform similarly. Also, the horizontal lines deform periodically over *x* with the deflections equal to zero at the midpoints of each length-*L* segment. As a result, each vertical beam can be independently analysed as an axially loaded vertical beam supported at a spacing of *L* along the height of the beam by horizontal beams of length *L* which are welded to the vertical beam at their centres. The horizontal beams can be considered to be simply supported at both ends due to zero curvature but free to translate horizontally. As the buckling wavelength (along *y*) approaches infinity, the distance between horizontal beams become smaller with respect to the wavelength, and thus, the angular stiffening effect of the horizontal beams can be estimated by analogy to an axially loaded vertical beam on a foundation of distributed rotational springs. Electronic supplementary material, appendix B details the differential relations governing the instability of an axially loaded beam on a distributed rotational spring foundation of intensity *K*_*t*_ (with the unit of moment per radian per unit length and dimension [N]), where it is shown that the critical compressive buckling load equals *P*_cr_=*K*_*t*_.

The goal here is therefore to obtain an equivalent rotational stiffness for the horizontal segments which are supporting vertical beams using the beam-column method. Once the effective rotational stiffness, *K*_*t*_, is obtained, the buckling load can be calculated using *P*_cr_=*K*_*t*_. Figure 2*c* shows a free-body diagram of the RVE, where the central node is rotated by angle *β* and the vertical beam segment of length *L* has an equivalent rotation of *α*, as resisted by the moment *M* in the middle as shown in the figure. Since each half-beam has a moment-free end, the set of beam-column relations for the RVE is *α*−*β*=(*ML*/(4*EI*))*Ψ(*q*_*y*_) and *β*=(*ML*/(4*EI*))*Ψ(*q*_*x*_), where $q_{x}=L/2*\sqrt{P_{x}/EI}$ and $q_{y}=L/2*\sqrt{P_{y}/EI}$. The equivalent rotational stiffness of the horizontal segments due to the swaying angle *α* can therefore be calculated as

4.6 $$K_{t}=\frac{M}{L\alpha }=\frac{4EI}{L^{2}}\frac{1}{\Psi (q_{x})+\Psi (q_{y})}.$$

Substituting into the *P*_cr_=*K*_*t*_ identity, the relations between parameters *q*_*x*_ and *q*_*y*_ needed for the long-wave instability of square grid based on the two variations along *x* and *y* are

4.7 $$\begin{array}{rl} & q_{x}^{2}(\Psi (q_{x})+\Psi (q_{y}))=1\\ \mathrm{and}\; & q_{y}^{2}(\Psi (q_{x})+\Psi (q_{y}))=1. \end{array}\}$$

These are mathematically identical to the relations obtained in equations (4.3) for buckling of the square grid according to the *x* or *y* swaying mode of instability. This approaching of the long-wave buckling strength to the swaying mode strength can also be justified from the physical point of view. By increasing the buckling wavelength in an unbounded structure (figure 2*c*), the deformation field corresponding to an arbitrary volume of *n*×*n* cells (n<∞) approaches the uniform deformation field observed in the swaying mode of buckling.

#### (iv) Results

Figure 3*a* shows the buckling strength curves corresponding to the *x* and *y* swaying modes, and the non-swaying buckling mode, in a square honeycomb under biaxial loading parallel to material principal directions (i.e. *x* and *y*). The horizontal and vertical axes correspond to normalized normal stresses in *x* and *y*, respectively, according to $\bar{\sigma }=(\sigma /E)/{\left(t/L\right)}^{3}$. The green curve denotes the non-swaying mode of buckling. The red lines correspond to the swaying microscopic buckling mode (equally, the long-wave buckling mode). The equi-biaxial buckling strength of a square grid is estimated as ${\bar{S}}_{c}^{x}={\bar{S}}_{c}^{y}=0.453$. The results are verified by the FE Eigenvalue analysis preformed at full as well as RVE scales.

Since three loading variables, *P*_*x*_, *P*_*y*_ and *P*_*xy*_, define the general in-plane loading, the results can be plotted in three dimensions for an arbitrary macroscopic state of stress. In figure 3*b*, the buckling surface of the square honeycomb is presented, which is described by the inner envelope of buckling stresses corresponding to the two rotational variations of the swaying or long-wave modes of buckling identically given by equations (4.4). The left- and right-hand sides of the instability surface in this figure correspond to the sway of horizontal and vertical beams in the square grid, respectively. The Eigenvalue instability in a square grid is independent of the value of shear loading, *P*_*xy*_, since the shear component does not explicitly appear in equations (4.4). However, sufficiently large values of shear stress can suppress the bifurcation response in a biaxially loaded square grid parallel to material principal directions (i.e. *x* and *y*), since the shear load component yields non-zero [*B*] matrices in equations (4.2) and (4.5). The macroscopic stress states corresponding to [*B*]=0 are also marked in this figure by dashed lines, sufficiently large deviations from which would cause the structure to deform according to a static, stable shear.

### (b) Triangular honeycombs

A similar approach was used to explore the buckling of triangular honeycomb. Differences occur in the number of beams in the RVE, and the possibility of two kinds of buckling pattern. Wang & McDowell [52] approximated the buckling strengths of a series of common cellular structures by means of a simplistic approach involving the equivalent beam length for cell walls of different periodic structures. They estimated the uniaxial buckling strength of triangular grid along any of the three cell wall directions to be $S_{c}/E_{s}=\left(2\pi^{2}/3\sqrt{3}\right)*{\left(t/L\right)}^{3}$. Similar to the case of a square honeycomb, this is an upper bound estimate of the actual buckling strength since it suppresses the rotations of the end nodes of the cell walls during buckling. More recently, Fan *et al.* [48] obtained a more precise estimation of uniaxial buckling strength of triangular honeycombs using the beam-column approach which allows for the rotation of the end nodes of cell wall during buckling. They expressed the uniaxial buckling strength along the cell wall direction (*x*) and the perpendicular to cell walls (*y*) as $S_{c}^{x}/E_{s}=2.543*{\left(t/L\right)}^{3}$ and $S_{c}^{y}/E_{s}=2.876*{\left(t/L\right)}^{3}$, respectively. Here, for the first time we provide a simple formula for the in-plane buckling of triangular grid under a general stress state.

#### (i) Mode I

For the sake of simplicity and also symmetrical results, the general state of in-plane macroscopic stress is uniquely expressed in terms of its normal lattice-direction components, *σ*_*aa*_, *σ*_*bb*_, *σ*_*cc*_, in the three in-plane material directions *a*=0^°^, *b*=120^°^ and *c*=240^°^ (measured from the *x*-axis) as shown in figure 1. Given these lattice-normal components of general stress tensor, the macroscopic *xy* stress tensor can be written as [55]

4.8 $$\left[\begin{array}{cc} \sigma_{xx} & \tau_{xy}\\ \tau_{xy} & \sigma_{yy} \end{array}\right]=\left[\begin{array}{cc} \sigma_{aa} & \frac{\sigma_{cc}}{\sqrt{3}}-\frac{\sigma_{bb}}{\sqrt{3}}\\ \frac{\sigma_{cc}}{\sqrt{3}}-\frac{\sigma_{bb}}{\sqrt{3}} & \frac{2\sigma_{cc}+2\sigma_{bb}-\sigma_{aa}}{3} \end{array}\right].$$

For example, uniaxial loadings *σ*_*xx*_=*σ* and *σ*_*yy*_=*σ* are represented by sets of (*σ*_*a*_, *σ*_*b*_, *σ*_*c*_) stresses equal to (*σ*, *σ*/4, *σ*/4) and (0,3*σ*/4,3*σ*/4), respectively.

**Figure 4.**
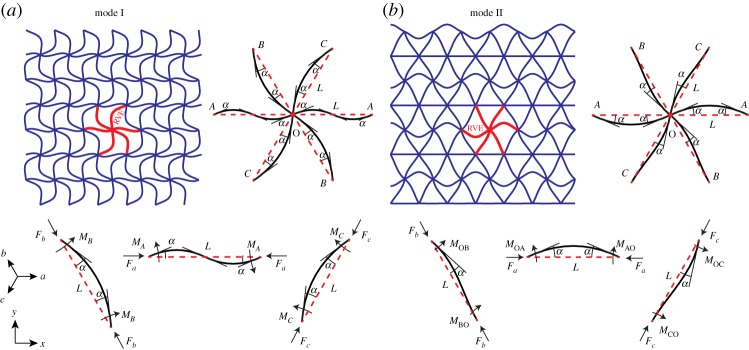
(*a*,*b*) Modes I and II of buckling in triangular honeycomb, respectively. In mode I, beam types OB and OC have opposite moments at their ends, while beam types OA have the same moments at their ends. In mode II, beam types OB and OC have zero rotation at one end, while beam types OA have opposite moments at their ends. The RVE for each mode is denoted by bold lines. Notations for the beam and nodal rotation in the RVE are given in the top right for each mode. Free-body diagrams of the RVE beam-elements are given in the bottom. (Online version in colour.)

Based on FE computations, only the two buckling modes indicated as I and II in figure 4 appear in the triangular grid structure under various loading conditions. Mode I is characterized by the equal rotation of all nodes in a row (e.g. along *a*), while adjacent rows have opposite rotations. Mode II is distinguished by zero rotation of nodes and beams in every other row of the structure and the alternating rotation of adjacent nodes in the remaining rows. Note that mode shapes I and II shown in figure 4 are unique with respect to direction *a* and symmetric relative to *b* and *c* directions, so the entire collapse surface is defined by equivalent mode shapes along the *a*, *b* and *c* directions.

Wang & McDowell [50] provided the internal axial forces per unit depth (positive when compressive) of beams in the equilateral triangular cell (isogrid) honeycomb of beam length *L*

4.9 $$\left[\begin{array}{c} F_{a}\\ F_{b}\\ F_{c} \end{array}\right]=-L\left[\begin{array}{ccc} \sqrt{3}/2 & -\sqrt{3}/6 & 0\\ 0 & \sqrt{3}/3 & -1\\ 0 & \sqrt{3}/3 & 1 \end{array}\right]\;\left[\begin{array}{c} \sigma_{xx}\\ \sigma_{yy}\\ \tau_{xy} \end{array}\right],$$

substituting from equation (4.8), we find the non-dimensional axial loading parameters $q_{a}=L\sqrt{F_{a}/(EI)}$, $q_{b}=L\sqrt{F_{b}/(EI)}$, $q_{c}=L\sqrt{F_{c}/(EI)}$ for the beams oriented along the *a*, *b* and *c* directions as follows:

4.10 $$q_{a}=\frac{2\text{i}\sqrt{5{\bar{\sigma }}_{aa}-{\bar{\sigma }}_{bb}-{\bar{\sigma }}_{cc}}}{3^{1/4}},q_{b}=\frac{2\text{i}\sqrt{5{\bar{\sigma }}_{bb}-{\bar{\sigma }}_{aa}-{\bar{\sigma }}_{cc}}}{3^{1/4}},q_{c}=\frac{2\text{i}\sqrt{5{\bar{\sigma }}_{cc}-{\bar{\sigma }}_{aa}-{\bar{\sigma }}_{bb}}}{3^{1/4}}.$$

Here, stresses are normalized according to $\bar{\sigma }=(\sigma /E)/{\left(t/L\right)}^{3}$. Since a triangular grid of inextensible beams can resolve all macroscopic loads with purely axial forces, load-induced beam transverse forces in this stretching dominated grid may be taken as zero.

The free-body diagram of a RVE for mode I buckling in a triangular grid is shown in figure 4*a*. In this mode, all nodes in a row rotate by the same angle. The *a* beams therefore have the same moment and same angle at each end, so the beam-column relations for each end are identical and only one is needed. Similarly, the *b* and *c* beams are symmetrically deformed with opposite moments and opposite relative angles, so the beam-column relations for each end are also identical (apart from a sign change). Beam-column and equilibrium relations for all three types of beam can be expressed in the following matrix form, where the first three rows are the three single beam-column relations for beams OA, OB and OC, and the last row expresses moment equilibrium for the central node O

4.11 $$\left[\begin{array}{cccc} 1 & -(\Psi (q_{a})-\Phi (q_{a})) & 0 & 0\\ 1 & 0 & -(\Psi (q_{b})+\Phi (q_{b})) & 0\\ 1 & 0 & 0 & -(\Psi (q_{c})+\Phi (q_{c}))\\ 0 & 1 & 1 & 1 \end{array}\right]\;\left[\begin{array}{c} \alpha \\ \frac{M_{A}L}{EI}\\ \frac{M_{B}L}{EI}\\ \frac{M_{C}L}{EI} \end{array}\right]=\left[\begin{array}{c} 0\\ 0\\ 0\\ 0 \end{array}\right].$$

Equating the determinant of the characteristic matrix to zero, the relation governing mode I buckling can be obtained as

4.12a $$q_{b}\cot\left(\frac{q_{b}}{2}\right)+q_{c}\cot\left(\frac{q_{c}}{2}\right)+\frac{q_{a}^{2}}{2-q_{a}\cot(q_{a}/2)}=0.$$

Similar expressions for the other two directions can be obtained by cyclically interchanging the subscripts:

4.12b $$q_{c}\cot\left(\frac{q_{c}}{2}\right)+q_{a}\cot\left(\frac{q_{a}}{2}\right)+\frac{q_{b}^{2}}{2-q_{b}\cot(q_{b}/2)}=0$$

and

4.12c $$q_{a}\cot\left(\frac{q_{a}}{2}\right)+q_{b}\cot\left(\frac{q_{b}}{2}\right)+\frac{q_{c}^{2}}{2-q_{c}\cot(q_{c}/2)}=0.$$

The buckling modes described by equations (4.12*a*–*c*) correspond to zero curvature at the midpoints of beams along the *a*, *b* and *c* directions, respectively.

#### (ii) Mode II

Figure 4*b* shows the RVE free-body diagram for mode II buckling. In this mode, alternate *a* lines remain straight during the buckling. The set of beam-column and equilibrium equation can be written in the following matrix form, where the first five rows represent the beam-column relations for beams OA, OB and OC, and the last row satisfies the equilibrium of moments around node O

4.13 $$\left[\begin{array}{cccccc} 1 & -\Psi (q_{a})-\Phi (q_{a}) & 0 & 0 & 0 & 0\\ 1 & 0 & -\Psi (q_{b}) & -\Phi (q_{b}) & 0 & 0\\ 0 & 0 & \Phi (q_{b}) & \Psi (q_{b}) & 0 & 0\\ 1 & 0 & 0 & 0 & -\Psi (q_{c}) & -\Phi (q_{c})\\ 0 & 0 & 0 & 0 & \Phi (q_{c}) & \Psi (q_{c})\\ 0 & 1 & 1 & 0 & 1 & 0 \end{array}\right]\;\left[\begin{array}{c} \alpha \\ \frac{M_{\mathrm{OA}}L}{EI}\\ \frac{M_{\mathrm{OB}}L}{EI}\\ \frac{M_{\mathrm{BO}}L}{EI}\\ \frac{M_{\mathrm{OC}}L}{EI}\\ \frac{M_{\mathrm{CO}}L}{EI} \end{array}\right]=\left[\begin{array}{c} 0\\ 0\\ 0\\ 0\\ 0\\ 0 \end{array}\right].$$

Equating the determinant of the characteristic matrix to zero, the relation between components of stress for mode II of buckling is

4.14a $$q_{a}\cot\left(\frac{q_{a}}{2}\right)+q_{b}\cot\left(\frac{q_{b}}{2}\right)\left(\frac{1-q_{b}\cot q_{b}}{2-q_{b}\cot(q_{b}/2)}\right)+q_{c}\cot\left(\frac{q_{c}}{2}\right)\left(\frac{1-q_{c}\cot q_{c}}{2-q_{c}\cot(q_{c}/2)}\right)=0,$$

and cyclically for *b* and *c* directions

4.14b $$q_{b}\cot\left(\frac{q_{b}}{2}\right)+q_{c}\cot\left(\frac{q_{c}}{2}\right)\left(\frac{1-q_{c}\cot q_{c}}{2-q_{c}\cot(q_{c}/2)}\right)+q_{a}\cot\left(\frac{q_{a}}{2}\right)\left(\frac{1-q_{a}\cot q_{a}}{2-q_{a}\cot(q_{a}/2)}\right)=0$$

and

4.14c $$q_{c}\cot\left(\frac{q_{c}}{2}\right)+q_{a}\cot\left(\frac{q_{a}}{2}\right)\left(\frac{1-q_{a}\cot q_{a}}{2-q_{a}\cot(q_{a}/2)}\right)+q_{b}\cot\left(\frac{q_{b}}{2}\right)\left(\frac{1-q_{b}\cot q_{b}}{2-q_{b}\cot(q_{b}/2)}\right)=0.$$

Geometrically, the three rotational variations of mode II buckling described by equations (4.14*a*–*c*) involve alternate straight beams along *a* (figure 4*b*), *b* and *c* directions in the buckled state, respectively. For the variation of mode II buckling shown in figure 4*b*, for example, the straightness of alternate horizontal type *a* beams is a special condition implying reflection symmetry about *x*- and *y*-axes, which requires, in effect, that the internal moment in those beams be small (i.e. *M*_BO_≅*M*_CO_). This condition is only satisfied when the characteristic matrix is symmetric with respect to *b* and *c* directions (i.e. *q*_b_=*q*_c_), corresponding to a biaxial state of loading in the principal material directions *x* and *y*. The necessity of a biaxial stress state for formation of mode II buckling in a triangular grid is also observed full-scale FE analysis, where the second mode of buckling described by equation (4.14*a*) is rapidly suppressed by adding the *τ*_*xy*_ shear stress component. Similarly, the two other variations of mode II with straight beams along *b* and *c* directions described by equations (4.14*b*,*c*) can only occur under microscopic biaxial loading along the *b* (and *b*⊥) and *c* (and *c*⊥) directions, respectively (the symbol ⊥ denotes perpendicular).

Under a general state of loading where no two loading parameters (i.e. *q*_*a*_, *q*_*b*_, *q*_*c*_) are equal, the macroscopic stresses required for the mode II of buckling given in equations (4.14) are equal or greater than those for the mode I of buckling given in equations (). Under a biaxial state of loading along material principal directions *x* and *y* (i.e. *q*_*b*_=*q*_*c*_), equation (4.14*a*) can be algebraically simplified to equations (4.12*b*,*c*). It can be shown numerically that under the *x*–*y* biaxial macroscopic loading with first principal stress along *y* (i.e. ${\bar{\sigma }}_{xx}<{\bar{\sigma }}_{yy}$), the first rotational variation of mode II buckling expressed by equation (4.14*a*), and the second and third rotations of mode I buckling expressed by equations (4.12*b*,*c*) equally yield the minimum required stress, and therefore are preferred buckling modes. For the case of *x*–*y* biaxial loading with the first principal stress along *x* (i.e. ${\bar{\sigma }}_{xx}>{\bar{\sigma }}_{yy}$), the first rotational variation of mode I buckling described by equation (4.12*a*) requires the minimum stress and is consequently preferred.

#### (iii) Results

**Figure 5.**
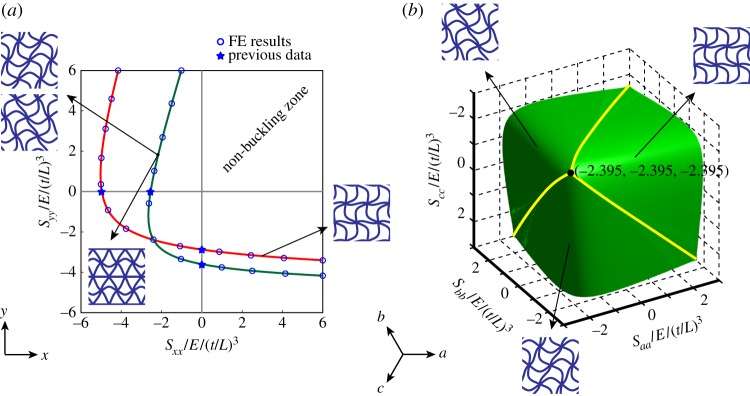
(*a*) Buckling of the triangular grid under *xy* biaxial loading according to modes I and II of buckling. (*b*) The buckling collapse surface of the triangular grid in the *abc* stress space, allowing prediction of buckling strength under a general in-plane state of stress. The three solid curved lines represent the stress states where more than one mode is possible (i.e. modes I and II). The three areas separated by the solid lines correspond to the three rotational variations of the primary buckling mode (i.e. mode I). (Online version in colour.)

Figure 5*a* shows the curves corresponding to the two modes of buckling under biaxial state of loading, where horizontal and vertical axes correspond to normalized normal stresses in *x* and *y* directions. The red line denotes the stress state corresponding to mode I buckling along *a*. The green line corresponds to mode II as well as the two rotations of mode II occurring along *b* or *c* directions. The green and red lines intersect at the equibiaxial state of macroscopic loading with the principal stresses equal to ${\bar{\sigma }}_{xx}={\bar{\sigma }}_{yy}=-2.395$. Under a state of pure shear described by compression along *x* and tension along *y* $\left({\bar{\sigma }}_{xx}=-{\bar{\sigma }}_{yy},{\bar{\sigma }}_{xx}<0\right)$, the buckling strength $\left(S_{c}^{x}\right)$ of triangular grid is given by $S_{c}^{x}/E_{s}=-2.099*{\left(t/L\right)}^{3}$. Also, under a state of pure shear described by compression along *y* and tension along *x* $\left({\bar{\sigma }}_{xx}=-{\bar{\sigma }}_{yy},{\bar{\sigma }}_{yy}<0\right)$, the buckling strength $\left(S_{c}^{y}\right)$ of triangular grid is $S_{c}^{y}/E_{s}=-3.213*{\left(t/L\right)}^{3}$. The results are verified by the FE Eigenvalue analysis preformed at the RVE scale, which is in agreement with full-scale FE results.

In figure 5*b*, the buckling surface corresponding to the triangular grid is presented, consisting of the inner envelope of buckling stresses corresponding to the three rotational variations of mode I of buckling given by equations (). The three solid curved lines in this figure correspond to the three biaxial states of loading along *x*–*y*, *x*–*z* and *y*–*z* directions where mode II buckling is also possible. The three areas separated by these solid curved lines correspond to the three rotational variations of mode I buckling characterized by double-curvature beams in the post-buckled state along *a*, *b* and *c* directions. Since the triangular grid is a stretching dominated structure, the condition [*B*]=0 is satisfied under all macroscopic stress states. Therefore, the entire instability surface plotted in figure 5*b* predicts an ideal bifurcation as discussed in §2.

### (c) Regular hexagonal honeycomb

**Figure 6.**
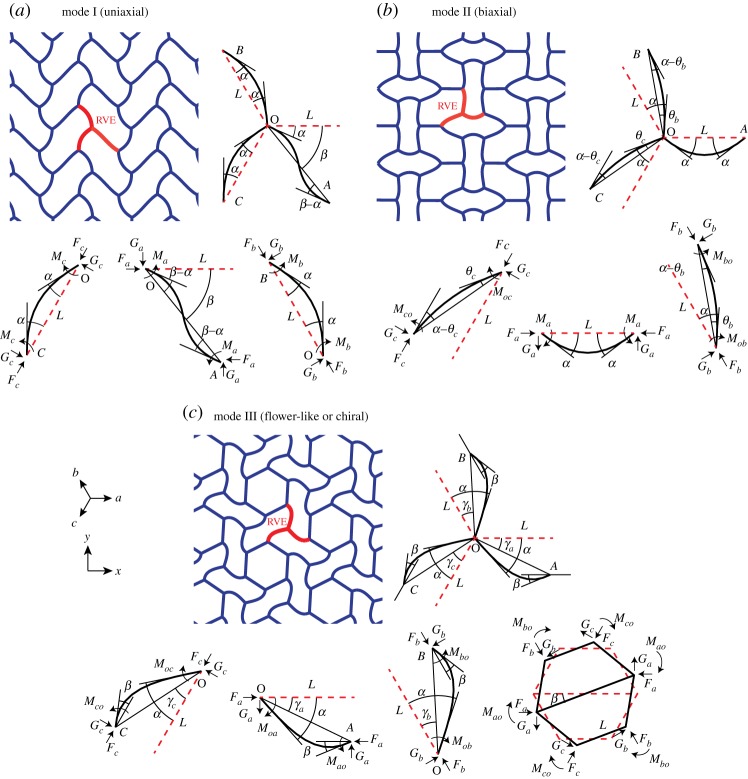
(*a*–*c*) Modes I, II and III of buckling observed in regular honeycomb, respectively. Modes I and II are characterized by a zigzag collapse of cells due to compression along the *x* direction and an alternating cell collapse due to compression perpendicular to the *x* direction, respectively. Mode III is a chiral cell configuration where groups of six highly deformed cells surround almost intact central cells. The RVE for each mode is denoted by bold lines. Notations for the beam and nodal rotation in the RVE for each mode are given in the top right. Free-body diagrams of the RVE beam-elements are given in the bottom. (Online version in colour.)

The nonlinear elastic response of regular hexagonal honeycomb has been previously studied by different researchers [23,24,33,35,56–58]. Gibson & Ashby [42] estimated the uniaxial buckling strength of the regular honeycomb structure in the cell wall direction (*x* in figure 1) as $S_{c}^{x}/E_{s}=0.22*{\left(t/L\right)}^{3}$, using the rotational spring approach. Later, Gibson *et al.* [35] used the beam-column relations to obtain the buckling strength of the hexagonal honeycomb structure under a biaxial state of loading parallel to *x* and *y* directions. They recognized two modes of buckling, commonly referred to as uniaxial and biaxial, for elastic bifurcation of a hexagonal honeycomb structure (figure 6*a*,*b*). The large deformation of cell edges before elastic buckling was taken in account by Zhang & Ashby [56] to analyse the biaxial in-plane buckling of hexagonal honeycombs. Ohno *et al.* numerically analysed the in-plane biaxial buckling of the regular hexagonal honeycomb using a homogenization framework of updated Lagrangian type [23]. Triantafyllidis & Schraad [24] studied the onset of bifurcation in hexagonal honeycombs under general in-plane loading using FE discretization of the Bloch wave theory. A third, more complex, flower-like mode, shown in figure 6*c*, is suggested for the buckling of regular hexagonal honeycomb structure [23]. This mode of buckling has been previously observed in experimental and numerical trials for hexagonal honeycombs with circular cells under equi-biaxial loading condition [28,47]. In this mode of buckling, groups of six highly deformed cells surround almost intact central cells, and the central cells rotate uniformly in either the clockwise or the counter-clockwise direction. Okumera *et al.* [58] later showed that the flower-like mode does not occur as the first bifurcation under macroscopic biaxial compression control. Here, for the first time we obtain expressions of buckling strength for the uniaxial, biaxial and flower-like modes of buckling of the hexagonal honeycomb structure under a general stress state.

#### (i) Mode I (uniaxial)

Because of the minimum threefold symmetry of regular honeycomb structure, the general state of in-plane macroscopic stress is expressed in terms of its normal components, *σ*_*aa*_, *σ*_*bb*_, *σ*_*cc*_, in the three in-plane material directions *a*=0^°^, *b*=120^°^ and *c*=240^°^ from the *x*-axis as shown in figure 1. The macroscopic *xy* stress tensor can be expressed in terms of the normal components of stress in *a*, *b* and *c* directions according to equation (4.8). Also the axial forces per unit depth (positive when compressive) in the cell walls oriented along *a*, *b*, *c* directions can be expressed as [1]

4.15 $$\left[\begin{array}{c} F_{a}\\ F_{b}\\ F_{c} \end{array}\right]=-L\left[\begin{array}{ccc} \sqrt{3} & 0 & 0\\ 0 & \sqrt{3} & 0\\ 0 & 0 & \sqrt{3} \end{array}\right]\;\left[\begin{array}{c} \sigma_{aa}\\ \sigma_{bb}\\ \sigma_{cc} \end{array}\right],$$

where *L* is the size of the hexagon side. The non-dimensional parameters *q*_*a*_, *q*_*b*_ and *q*_*c*_, are therefore found as $q_{a}=L\sqrt{F_{a}/(EI)}=2\text{i}\sqrt{3\sqrt{3}{\bar{\sigma }}_{aa}}$ and cyclically for *q*_*b*_ and *q*_*c*_. Also the transverse forces in the cell walls of the hexagonal honeycomb can be obtained according to [55]

4.16 $$\left[\begin{array}{c} G_{a}\\ G_{b}\\ G_{c} \end{array}\right]=\frac{1}{\sqrt{3}}\left[\begin{array}{ccc} 0 & 1 & -1\\ -1 & 0 & 1\\ 1 & -1 & 0 \end{array}\right]\;\left[\begin{array}{c} F_{a}\\ F_{b}\\ F_{c} \end{array}\right].$$

For mode I (uniaxial) buckling, the structural RVE shown in figure 6*a* consists of the three beams OA, OB and OC oriented along *a*, *b* and *c* directions, respectively. The pre- and post-buckling configurations of the RVE are shown by red dashed lines and solid black lines, respectively. Taking into account the symmetry requirements in the buckled configuration of the structure, beam OA is under equal end moments, denoted by *M*_*a*_, and beams OB and OC each are under opposite (with equal magnitude) end moments denoted by *M*_*b*_ and *M*_*c*_, respectively, as shown in figure 6*a*. The set of beam-column and equilibrium relations of the three different bar types OA, OB and OC can be expressed in the following matrix form, where the first three matrix rows represent the beam-column relations for beams OA, OB and OC, the fourth line corresponds to equilibrium of node O, and the last relation expresses moment equilibrium in beam OA around node O.

4.17 $$\left[\begin{array}{ccccc} -\Psi (q_{a})+\Phi (q_{a}) & 0 & 0 & -1 & 1\\ 0 & -\Psi (q_{b})-\Phi (q_{b}) & 0 & 1 & 0\\ 0 & 0 & -\Psi (q_{c})-\Phi (q_{c}) & 1 & 0\\ 1 & -1 & -1 & 0 & 0\\ 2 & 0 & 0 & 0 & -q_{a}^{2} \end{array}\right]\left[\begin{array}{c} \frac{M_{a}L}{EI}\\ \frac{M_{b}L}{EI}\\ \frac{M_{c}L}{EI}\\ \alpha \\ \beta \end{array}\right]=\;\left[\begin{array}{c} 0\\ 0\\ 0\\ 0\\ -\frac{G_{a}L^{2}}{EI} \end{array}\right]\;.$$

Using the symbolic toolbox in MATLAB to set |*A*|=0, the relation between *q*_*a*_, *q*_*b*_ and *q*_*c*_ expressing the mode I instability of a regular honeycomb structure under a general loading is

4.18 $$q_{a}\tan\left(\frac{q_{a}}{2}\right)-q_{b}\cot\left(\frac{q_{b}}{2}\right)-q_{c}\cot\left(\frac{q_{c}}{2}\right)=0.$$

Therefore, the expression of mode I instability in the *abc* stress space would be

4.19a $$\sqrt{{\bar{\sigma }}_{aa}}\;\text{tanh}\left(\sqrt{3\sqrt{3}{\bar{\sigma }}_{aa}}\right)+\sqrt{{\bar{\sigma }}_{bb}}\;\text{coth}\left(\sqrt{3\sqrt{3}{\bar{\sigma }}_{bb}}\right)+\sqrt{{\bar{\sigma }}_{cc}}\;\text{coth}\left(\sqrt{3\sqrt{3}{\bar{\sigma }}_{cc}}\right)=0,$$

where the bar above the stresses means they are normalized according to $\bar{\sigma }=(\sigma /E)/{\left(t/L\right)}^{3}$. Note that the uniaxial mode of buckling shown in figure 6*a* does not possess the threefold symmetry observed in the honeycomb lattice (i.e. the rotational symmetry with respect to the three directions *a*, *b* and *c*), and therefore the following additional relations are needed to describe the buckling strength of a regular honeycomb according to uniaxial modes of buckling along *b* and *c* directions

4.19b $$\sqrt{{\bar{\sigma }}_{bb}}\;\text{tanh}\left(\sqrt{3\sqrt{3}{\bar{\sigma }}_{bb}}\right)+\sqrt{{\bar{\sigma }}_{cc}}\;\text{coth}\left(\sqrt{3\sqrt{3}{\bar{\sigma }}_{cc}}\right)+\sqrt{{\bar{\sigma }}_{aa}}\;\text{coth}\left(\sqrt{3\sqrt{3}{\bar{\sigma }}_{aa}}\right)=0$$

and

4.19c $$\sqrt{{\bar{\sigma }}_{cc}}\;\text{tanh}\left(\sqrt{3\sqrt{3}{\bar{\sigma }}_{cc}}\right)+\sqrt{{\bar{\sigma }}_{aa}}\;\text{coth}\left(\sqrt{3\sqrt{3}{\bar{\sigma }}_{aa}}\right)+\sqrt{{\bar{\sigma }}_{bb}}\;\text{coth}\left(\sqrt{3\sqrt{3}{\bar{\sigma }}_{bb}}\right)=0.$$

From the geometrical point of view, the buckling modes described by equations (4.19) correspond to zero curvature at midpoints of beams along the *a*, *b* and *c* directions, respectively. Substituting *q*_*b*_=*q*_*c*_ in equations (4.19), the result given in [35] for the instability of a regular hexagonal honeycomb under the simplified case of *x*–*y* biaxial loading condition is obtained.

#### (ii) Mode II (biaxial)

The biaxial mode of buckling and the associated RVE are shown in figure 6*b*. According to symmetry requirements the beam *OA* is under opposite (with equal magnitude) moments, denoted by *M*_*a*_, at the ends. Beams OB and OC are subjected to end moments *M*_*ob*_, *M*_*bo*_, *M*_*oc*_, *M*_*co*_, as shown in the figure. The set of beam-column and equilibrium relations of the three different bar types OA, OB and OC can be expressed in the following matrix form, where the first five matrix rows represent the beam-column relations for beams OA, OB and OC, the sixth line corresponds to moment equilibrium of node O, and the last two relations satisfy the moment equilibrium in beams OB and OC about node O.

4.20 $$\begin{array}{rl} & \left[\begin{array}{cccccccc} -\Phi (q_{a})-\Psi (q_{a}) & 0 & 0 & 0 & 0 & 1 & 0 & 0\\ 0 & -\Psi (q_{b}) & -\Phi (q_{b}) & 0 & 0 & 0 & 1 & 0\\ 0 & -\Phi (q_{b}) & -\Psi (q_{b}) & 0 & 0 & 1 & -1 & 0\\ 0 & 0 & 0 & -\Psi (q_{c}) & -\Phi (q_{c}) & 0 & 0 & 1\\ 0 & 0 & 0 & -\Phi (q_{c}) & -\Psi (q_{c}) & 1 & 0 & -1\\ 1 & 1 & 0 & 1 & 0 & 0 & 0 & 0\\ 0 & 1 & -1 & 0 & 0 & q_{b}^{2} & -q_{b}^{2} & 0\\ 0 & 0 & 0 & 1 & -1 & q_{c}^{2} & 0 & -q_{c}^{2} \end{array}\right]\;\left[\begin{array}{c} M_{a}L/(EI)\\ M_{ob}L/(EI)\\ M_{bo}L/(EI)\\ M_{oc}L/(EI)\\ M_{co}L/(EI)\\ \alpha \\ \theta_{b}\\ \theta_{c} \end{array}\right]\\ & \;=\left[\begin{array}{c} 0\\ 0\\ 0\\ 0\\ 0\\ 0\\ G_{b}L^{2}/(EI)\\ G_{c}L^{2}/(EI) \end{array}\right]. \end{array}$$

Using the symbolic toolbox in MATLAB to set |*A*|=0, the relation between *q*_*a*_, *q*_*b*_ and *q*_*c*_ expressing the instability of a regular honeycomb structure in mode II under a general loading is

4.21 $$q_{a}\cot\left(\frac{q_{a}}{2}\right)+q_{b}\cot(q_{b})+q_{c}\cot(q_{c})=0,$$

or considering all three rotations corresponding to this mode in the *abc* stress space

4.22a $$\begin{array}{rl} & \sqrt{{\bar{\sigma }}_{aa}}\;\text{coth}\left(\sqrt{3\sqrt{3}{\bar{\sigma }}_{aa}}\right)+\sqrt{{\bar{\sigma }}_{bb}}\;\text{coth}\left(2\sqrt{3\sqrt{3}{\bar{\sigma }}_{bb}}\right)+\sqrt{{\bar{\sigma }}_{cc}}\;\text{coth}\left(2\sqrt{3\sqrt{3}{\bar{\sigma }}_{cc}}\right)=0, \end{array}$$

4.22b $$\begin{array}{rl} & \sqrt{{\bar{\sigma }}_{bb}}\;\text{coth}\left(\sqrt{3\sqrt{3}{\bar{\sigma }}_{bb}}\right)+\sqrt{{\bar{\sigma }}_{cc}}\;\text{coth}\left(2\sqrt{3\sqrt{3}{\bar{\sigma }}_{cc}}\right)+\sqrt{{\bar{\sigma }}_{aa}}\;\text{coth}\left(2\sqrt{3\sqrt{3}{\bar{\sigma }}_{aa}}\right)=0, \end{array}$$

and

4.22c $$\begin{array}{rl} & \sqrt{{\bar{\sigma }}_{cc}}\;\text{coth}\left(\sqrt{3\sqrt{3}{\bar{\sigma }}_{cc}}\right)+\sqrt{{\bar{\sigma }}_{aa}}\;\text{coth}\left(2\sqrt{3\sqrt{3}{\bar{\sigma }}_{aa}}\right)+\sqrt{{\bar{\sigma }}_{bb}}\;\text{coth}\left(2\sqrt{3\sqrt{3}{\bar{\sigma }}_{bb}}\right)=0, \end{array}$$

Geometrically, the buckling modes described by equations (4.22*a*–*c*) include straight beams in the post-buckled configuration along *a*, *b* and *c* directions, respectively.

The zero curvature in a series of horizontal beams in the post-buckled state shown in figure 6*b* requires the internal moment throughout those beams to be zero. Noting that the two pairs of opposite moments *M*_*bo*_ and *M*_*co*_ act on the ends of these beams, the last condition requires *M*_*bo*_=*M*_*co*_. This is only satisfied when the characteristic matrix is symmetrical with respect to *b* and *c* directions, which translates to a biaxial state of loading in the principal material directions *x* and *y*. This is supported by full-scale FE analysis, where the mode II buckling is observed under *x*–*y* biaxial loading, and is rapidly suppressed by increasing the magnitude of macroscopic *xy* shear stress component. Given a biaxial state of macroscopic stress, equations (4.22) can be simplified to the result obtained in [35] for the instability of regular honeycomb under the simple case of *x*–*y* biaxial loading condition.

#### (iii) Mode III (flower-like or chiral)

For the flower-like mode of buckling shown in figure 6*c*, the set of beam-column and equilibrium relations of three different bar types OA, OB and OC can be expressed in the following matrix form, where the first and the second rows satisfy the moment equilibrium of node O and the intact central hexagon, respectively; rows three to five satisfy the moment equilibrium of beams OA, OB and OC about O; and rows six to eleven represent the beam-column relations for beams OA, OB and OC.

4.23 $$\begin{array}{rl} & \left[\begin{array}{ccccccccccc} 1 & 1 & 1 & 0 & 0 & 0 & 0 & 0 & 0 & 0 & 0\\ 0 & 0 & 0 & 1 & 1 & 1 & 0 & -(q_{a}^{2}+q_{b}^{2}+q_{c}^{2}) & 0 & 0 & 0\\ 1 & 0 & 0 & -1 & 0 & 0 & 0 & 0 & q_{a}^{2} & 0 & 0\\ 0 & 1 & 0 & 0 & -1 & 0 & 0 & 0 & 0 & q_{b}^{2} & 0\\ 0 & 0 & 1 & 0 & 0 & -1 & 0 & 0 & 0 & 0 & q_{c}^{2}\\ \Psi (q_{a}) & 0 & 0 & \Phi (q_{a}) & 0 & 0 & -1 & 0 & 1 & 0 & 0\\ 0 & \Psi (q_{b}) & 0 & 0 & \Phi (q_{b}) & 0 & -1 & 0 & 0 & 1 & 0\\ 0 & 0 & \Psi (q_{c}) & 0 & 0 & \Phi (q_{c}) & -1 & 0 & 0 & 0 & 1\\ \Phi (q_{a}) & 0 & 0 & \Psi (q_{a}) & 0 & 0 & 0 & -1 & -1 & 0 & 0\\ 0 & \Phi (q_{b}) & 0 & 0 & \Psi (q_{b}) & 0 & 0 & -1 & 0 & -1 & 0\\ 0 & 0 & \Phi (q_{c}) & 0 & 0 & \Psi (q_{c}) & 0 & -1 & 0 & 0 & -1 \end{array}\right]\\ & \;\times \left[\begin{array}{c} M_{oa}L/(\text{EI})\\ M_{ob}L/(\text{EI})\\ M_{oc}L/(\text{EI})\\ M_{ao}L/(\text{EI})\\ M_{bo}L/(\text{EI})\\ M_{co}L/(\text{EI})\\ \alpha \\ \beta \\ \gamma_{a}\\ \gamma_{b}\\ \gamma_{c} \end{array}\right]=\left[\begin{array}{c} 0\\ \left(G_{a}+G_{b}+G_{c})L^{2}/(\text{EI}\right)\\ G_{a}L^{2}/(\text{EI})\\ G_{b}L^{2}/(\text{EI})\\ G_{c}L^{2}/(\text{EI})\\ 0\\ 0\\ 0\\ 0\\ 0\\ 0 \end{array}\right]. \end{array}$$

Equating the determinant of the characteristic matrix to zero yields the following relation between *q*_*a*_, *q*_*b*_ and *q*_*c*_ for flower-like instability of a regular hexagonal honeycomb

4.24 $$\begin{array}{rl} & \frac{2}{q_{a}^{2}+q_{b}^{2}+q_{c}^{2}}+\frac{1}{q_{a}\tan(q_{a}/2)+q_{b}\tan(q_{b}/2)+q_{c}\tan(q_{c}/2)}\\ & \;=\frac{1}{q_{a}\cot(q_{a}/2)+q_{b}cot(q_{b}/2)+q_{c}\cot(q_{c}/2)}, \end{array}$$

where $q_{a}=2\text{i}\sqrt{3\sqrt{3}{\bar{\sigma }}_{aa}}$ and cyclically for *q*_*b*_ and *q*_*c*_.

#### (iv) Results

For all loading directions, each defined by a ratio between components of macroscopic stress, the stresses required for biaxial mode of buckling described by equations (4.22) are equal or greater than those needed for the uniaxial mode of buckling given by equations (4.19). Under in-plane *x*–*y* biaxial loading (i.e. ${\bar{\tau }}_{xy}=0$ or equivalently ${\bar{\sigma }}_{bb}={\bar{\sigma }}_{cc}$), equations (4.19*b*,*c*) can be simplified to equation (4.22*a*) using the trigonometric identity coth(2*x*)=( coth(*x*)+ tanh(*x*))/2. For a regular honeycomb structure under biaxial macroscopic loading with the first principal stress along *x* (i.e. ${\bar{\sigma }}_{xx}>{\bar{\sigma }}_{yy}$), the microscopic instability could arise through either the uniaxial mode with alternative swaying of beams along the *a* direction, or the biaxial mode involving straight beams along the *a* direction. In full-scale numerical trials, the buckling mode under this loading condition is determined by the boundary conditions applied to the finite-size FE model. For the case of biaxial loading with the first principal stress along *y* (i.e. ${\bar{\sigma }}_{xx}<{\bar{\sigma }}_{yy}$), the uniaxial mode of buckling is the preferred mode.

**Figure 7.**
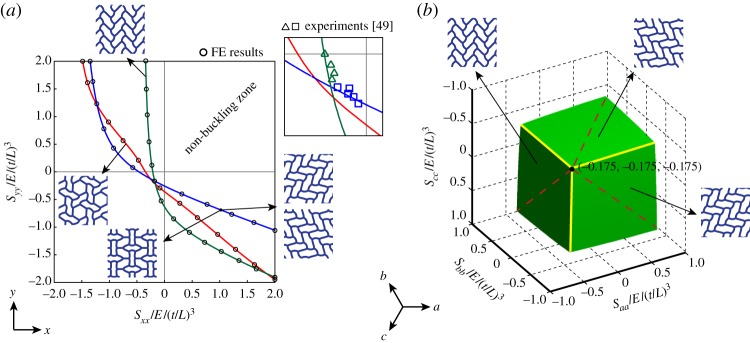
(*a*) Biaxial buckling collapse of regular hexagonal honeycomb under biaxial loading along *x* (the so-called armchair or ribbon direction) and *y* (the so-called zigzag or transverse direction) according to uniaxial, biaxial and flower-like modes of buckling. The inset shows comparison with the experimental data from [49]. (*b*) The buckling collapse surface of the regular honeycomb structure in the *abc* stress space, allowing prediction of buckling strength under a general in-plane state of stress. The three solid lines represent the stress states where more than one mode is possible (i.e. modes I and II). The three distinct areas on the buckling surface separated by the solid lines correspond to the three rotational variations of the primary buckling mode (i.e. mode I). The condition [*B*]=0 is marked by dashed lines on the instability surface. (Online version in colour.)

Figure 7*a* shows the curves corresponding to uniaxial, biaxial and flower-like modes of buckling in a regular honeycomb under biaxial state of loading, where horizontal and vertical axes correspond to normalized normal stresses in *x* and *y* directions, respectively, according to $\bar{\sigma }=(\sigma /E)/{\left(t/L\right)}^{3}$. The green and red lines denote the uniaxial mode of buckling along *a* (or *x*) and the flower-like modes, respectively. The blue line corresponds to the biaxial mode as well as the two variations of the uniaxial mode occurring along *b* or *c* directions. The green and blue lines intersect at the equi-biaxial state of macroscopic loading with the principal stresses equal to ${\bar{\sigma }}_{xx}={\bar{\sigma }}_{yy}=-0.175$. The flower-like mode is not a dominant mode under any loading condition. However, the macroscopic stresses needed for this mode become relatively close to those of uniaxial and biaxial modes under equi-biaxial loading with the macroscopic stresses ${\bar{\sigma }}_{xx}={\bar{\sigma }}_{yy}=-0.198$. The results are verified by the FE Eigenvalue analyses preformed at the RVE scale, which are in good agreement with full-scale FE results. The inset of the figure shows a comparison of the analytical results with experimental data from [49]. In figure 7*b*, the buckling surface for a regular hexagonal honeycomb structure under arbitrary stress state is presented. The surface is mathematically described by the inner envelope of buckling stresses corresponding to the three rotational variations of the unixial mode (mode I) of buckling given by equations (4.19). The three edges marked by solid lines correspond to the three biaxial states of loading along *x*–*y*, *b* (and *b*⊥) and *c* (and *c*⊥) directions, where the biaxial mode (mode II) of buckling is also possible. The three areas of the instability surface separated by these lines correspond to the three rotational variations of mode I buckling. On each edge, macroscopic stress states corresponding to the condition [*B*]=0 in equation (4.17) are marked by dashed lines. Deviation from these lines would cause the structure to fold up in a stable way. Note that for a mode II buckling the condition [*B*]=0 in equation (4.20) is only satisfied under an equi-biaxial stress state.

## 5. Conclusion

Table 1 summarizes the closed-form relations for the buckling strength of regular, chiral and hierarchical hexagonal honeycombs and triangular and square grids (the periodic buckling modes and derivations for tri-chiral and hierarchical honeycombs are similar to the regular honeycomb structure and are given in the electronic supplementary material, appendix D).

**Table 1.** Relations describing the instability stress surface for the structures studied.

honeycomb type	instability stress surface $\left(\bar{\sigma }=(\sigma /E)/{\left(t/L\right)}^{3}\right)$	
square grid	$\begin{array}{rl} & {\bar{\sigma }}_{yy}\left(1-\sqrt{3{\bar{\sigma }}_{xx}}\;\text{coth}\left(\sqrt{3{\bar{\sigma }}_{xx}}\right)\right)-{\bar{\sigma }}_{xx}\left(\sqrt{3{\bar{\sigma }}_{yy}}\;\text{coth}\left(\sqrt{3{\bar{\sigma }}_{yy}}\right)\right)=0\\ & {\bar{\sigma }}_{xx}\left(1-\sqrt{3{\bar{\sigma }}_{yy}}\;\text{coth}\left(\sqrt{3{\bar{\sigma }}_{yy}}\right)\right)-{\bar{\sigma }}_{yy}\left(\sqrt{3{\bar{\sigma }}_{xx}}\;\text{coth}\left(\sqrt{3{\bar{\sigma }}_{xx}}\right)\right)=0 \end{array}\}$	
triangular grid	$\begin{array}{r} q_{b}\cot\left(\frac{q_{b}}{2}\right)+q_{c}\cot\left(\frac{q_{c}}{2}\right)+\frac{q_{a}^{2}}{2-q_{a}\cot(q_{a}/2)}=0\\ q_{c}\cot\left(\frac{q_{c}}{2}\right)+q_{a}\cot\left(\frac{q_{a}}{2}\right)+\frac{q_{b}^{2}}{2-q_{b}\cot(q_{b}/2)}=0\\ q_{a}\cot\left(\frac{q_{a}}{2}\right)+q_{b}\cot\left(\frac{q_{b}}{2}\right)+\frac{q_{c}^{2}}{2-q_{c}\cot(q_{c}/2)}=0 \end{array}\}$	
	$\left(q_{a}=\frac{2\text{i}\sqrt{5{\bar{\sigma }}_{aa}-{\bar{\sigma }}_{bb}-{\bar{\sigma }}_{cc}}}{3^{1/4}},q_{b}=\frac{2\text{i}\sqrt{5{\bar{\sigma }}_{bb}-{\bar{\sigma }}_{aa}-{\bar{\sigma }}_{cc}}}{3^{1/4}},q_{c}=\frac{2\text{i}\sqrt{5{\bar{\sigma }}_{cc}-{\bar{\sigma }}_{aa}-{\bar{\sigma }}_{bb}}}{3^{1/4}}\right)$	
	$\begin{array}{rl} \Delta_{a} & \equiv \sqrt{{\bar{\sigma }}_{aa}}\;\text{tanh}\left(\sqrt{3\sqrt{3}{\bar{\sigma }}_{aa}}\right)+\sqrt{{\bar{\sigma }}_{bb}}\;\text{coth}\;\left(\sqrt{3\sqrt{3}{\bar{\sigma }}_{bb}}\right)+\sqrt{{\bar{\sigma }}_{cc}}\;\text{coth}\left(\sqrt{3\sqrt{3}{\bar{\sigma }}_{cc}}\right)\\ \Delta_{b} & \equiv \sqrt{{\bar{\sigma }}_{bb}}\;\text{tanh}\left(\sqrt{3\sqrt{3}{\bar{\sigma }}_{bb}}\right)+\sqrt{{\bar{\sigma }}_{cc}}\;\text{coth}\;\left(\sqrt{3\sqrt{3}{\bar{\sigma }}_{cc}}\right)+\sqrt{{\bar{\sigma }}_{aa}}\;\text{coth}\left(\sqrt{3\sqrt{3}{\bar{\sigma }}_{aa}}\right)\\ \Delta_{c} & \equiv \sqrt{{\bar{\sigma }}_{cc}}\;\text{tanh}\left(\sqrt{3\sqrt{3}{\bar{\sigma }}_{cc}}\right)+\sqrt{{\bar{\sigma }}_{aa}}\;\text{coth}\left(\sqrt{3\sqrt{3}{\bar{\sigma }}_{aa}}\right)+\sqrt{{\bar{\sigma }}_{bb}}\;\text{coth}\left(\sqrt{3\sqrt{3}{\bar{\sigma }}_{bb}}\right) \end{array}$	
	regular	*Δ*_*a*_=0,*Δ*_*b*_=0,*Δ*_*c*_=0
hexagon based	tri-chiral (tan⁡θ≪1)	$\begin{array}{rl} & \sqrt{3\sqrt{3}}({\bar{\sigma }}_{a^{\prime }a^{\prime }}+{\bar{\sigma }}_{b^{\prime }b^{\prime }}+{\bar{\sigma }}_{c^{\prime }c^{\prime }})\tan^{2}\theta +\Delta_{a^{\prime }}=0\\ & \sqrt{3\sqrt{3}}({\bar{\sigma }}_{a^{\prime }a^{\prime }}+{\bar{\sigma }}_{b^{\prime }b^{\prime }}+{\bar{\sigma }}_{c^{\prime }c^{\prime }})\tan^{2}\theta +\Delta_{b^{\prime }}=0\\ & \sqrt{3\sqrt{3}}({\bar{\sigma }}_{a^{\prime }a^{\prime }}+{\bar{\sigma }}_{b^{\prime }b^{\prime }}+{\bar{\sigma }}_{c^{\prime }c^{\prime }})\tan^{2}\theta +\Delta_{c^{\prime }}=0 \end{array}\}$
	hierarchical (*γ*≪1)	$\begin{array}{r} ({\bar{\sigma }}_{aa}+{\bar{\sigma }}_{bb}+{\bar{\sigma }}_{cc})\frac{2\sqrt{3\sqrt{3}}\gamma }{1-2\gamma }+\Delta_{a}=0\\ ({\bar{\sigma }}_{aa}+{\bar{\sigma }}_{bb}+{\bar{\sigma }}_{cc})\frac{2\sqrt{3\sqrt{3}}\gamma }{1-2\gamma }+\Delta_{b}=0\\ ({\bar{\sigma }}_{aa}+{\bar{\sigma }}_{bb}+{\bar{\sigma }}_{cc})\frac{2\sqrt{3\sqrt{3}}\gamma }{1-2\gamma }+\Delta_{c}=0 \end{array}\}$

An interesting feature in buckling of hexagonal and triangular honeycombs is the possibility of secondary modes of buckling which are observed only under the *x*–*y* biaxial state of macroscopic stress. These secondary modes were shown to occur at the same macroscopic stress levels required by the primary modes of buckling under *x*–*y* biaxial stress state. Similarly, there is no difference between the closed-form strength relations corresponding to the swaying and the long-wave buckling patterns in a square grid when the wavelength in the long-wave buckling mode approaches infinity. The preferred buckling mode in these cases is controlled by the far-field boundary conditions, and by imperfections in large-scale FE models.

Use of the beam-column approach for calculating the buckling strength of cellular structures requires a caveat with regard to the effect of cell wall lateral loads (i.e. non-axial components of cell wall reaction force) on suppressing instability in periodic structures. These lateral load components, appearing on the right-hand side of equation (2.3) (i.e. vector *B*), eliminate the sudden collapse normally expected of buckling, and smooth out the associated bifurcation in the macroscopic load–displacement curve. As shown for a regular honeycomb structure under uniaxial *y*-compression, the lateral loads on the oblique beams cause the structure to fold up in a stable way [1]. This suppression of buckling happens for a square grid under pure macroscopic *xy* shear loading, where the characteristic matrix was shown to be independent of lateral loads in the vertical and horizontal beams, and thus of the amount of shear stress. As the value of shear stress increases compared with axial stresses, the beams simply deflect statically until the pre-buckling deformations become so large that they suppress buckling. For the triangular grid with a stretching dominated behaviour, however, the lateral reaction forces in the cell walls are essentially zero. As a result, the cell walls in the structure do not undergo a pre-buckling bending deformation, and bifurcation of the macroscopic load–displacement curve is observed under all stress states.

Regarding the post-buckling response of the regular, hierarchical and chiral honeycombs considered here, FE simulations on finite honeycomb models reveal a distinct sequence of deformed configuration; that is, linear elastic behaviour, weakly stable or unstable buckling followed by a significant restiffening caused by densification. This sequence has been previously reported in the crushing response of honeycombs and foams [28,32,59]. The buckling investigations presented, in conjunction with recent progress on plastic-collapse criteria [55], are making it possible to construct comprehensive multi-axial, multi-failure surfaces for cellular structures.
